# Were metabolic and other chronic diseases the driven onset epidemic forces of COVID-19 in Mexico?

**DOI:** 10.3389/fpubh.2023.995602

**Published:** 2023-08-07

**Authors:** Gerardo Acevedo-Sánchez, Gustavo Mora-Aguilera, Juan J. Coria-Contreras, Ikuri Álvarez-Maya

**Affiliations:** ^1^Laboratory of Epidemiological Risk Analysis (LANREF), Postgraduate College, Montecillo Campus, Texcoco, State of Mexico, Mexico; ^2^Center for Research and Applied Technology in Jalisco (CIATEJ), Guadalajara, Jalisco, Mexico

**Keywords:** SARS-CoV-2, comorbidities, mortality risk, infection risk, first wave

## Abstract

The underline hypothesis of this study was that SARS-CoV-2 can infect individuals regardless of health condition, sex, and age in opposition to the classical epidemiological assumption of an identifiable susceptible subpopulation for epidemic development. To address this issue, a population cohort with 24.4 million metadata associated with 226,089 official RT-qPCR positive and 283,450 negative cases, including 27,769 deceased, linked putatively to *B.1*. and *B.1.1*. SARS-CoV-2 lineages were analyzed. The analysis baseline was to determine the infection and mortality structure of the diseased cohort at the onset-exponential phase of the first epidemic wave in Mexico under the assumption of limited herd immunity. Individuals with nonchronic diseases (NOCDs) were compared with those exhibiting at least one of 10 chronic diseases (CDs) adjusted by age and sex. Risk factors for infection and mortality were estimated with classification and regression tree (CART) and cluster analysis based on Spearman's matrix of *rho*-values in RStudio^®^, complemented with two proposed *mortality indices*. SARS-CoV-2 infection was independent of health condition (52.8% NOCD *vs*. 47.2% CDs; *p* = 0.001–0.009) but influenced by age >46 in one risk analysis scenario (*p* < 0.001). Sex contributed 9.7% to the overall risk. The independent effect was supported by the health structure of negative cases with a similar tendency but a higher proportion of NOCDs (61.4%, *p* = 0.007). The infection probability in individuals with one CD was determined by the disease type and age, which was higher in those older individuals (≥56 years) exhibiting diabetes (12.3%, *cp* = 0.0006), hypertension (10.1%, *cp* < 0.0001), and obesity (7.8%, *cp* = 0.001). In contrast, the mortality risk was heavily influenced by CD conditioned by sex and age, accounting for 72.3% of total deaths (*p* = 0.001–0.008). Significant mortality risk (48%) was comprised of women and men (w, m) aged ≥56 years with diabetes (19% w and 27.9% m, *cp* < 0.0004), hypertension (11.5% w, *cp* = 0.0001), and CKD (3.5% w and 5.3% m, *cp* = 0.0009). Older people with diabetes and hypertension comorbidity increased the risk to 60.5% (*p* = 0.001). Based on a *mortality-weighted index*, women were more vulnerable to preexisting metabolic or cardiovascular diseases. These findings support our hypothesis and justify the need for surveillance systems at a communitarian level. This is the first study addressing this fundamental epidemiological question.

## Introduction

SARS-CoV-2, the most successful zoonotic coronavirus in human history, has caused over 668 million infection cases and more than 6.8 million deaths worldwide through several epidemic waves (1, 2). Since the Wuhan outbreak in China (3), at least 19 variants of the epidemic have emerged and spread rapidly before an effective natural immunological response (4). In infectious epidemic diseases, the classical paradigm behind the susceptible, infected, and recovered (SIR) individuals and any descriptive or predictive epidemiological model imply the preexistence of a susceptible subpopulation due to genetic, epigenetic, clinical, and environmental determinants as the driving forces for contagion (5–10). With COVID-19 epidemics, early findings supported that chronic diseases (CDs), age, and, to a less extent, sex were associated with the success and clinical outcomes of SARS-CoV-2 infection. However, most results were derived at the hospital level, from a small diagnostic dataset, or framed for descriptive epidemiological studies (3, 11, 12). More vital efforts should be addressed from the perspective of mechanistic epidemiology to enhance comprehensive prevention health systems to cope with the increasing risk of emerging and reemerging new human diseases. This study hypothesized that SARS-CoV-2 can infect individuals regardless of their health condition in opposition to the classical epidemiological assumption of an identifiable susceptible subpopulation for epidemic development. It was assumed that fast spreading, limited and unsteady immunological response toward a newly encountered pathogen, constrained clinical knowledge for treatment, and unprepared public health systems were fully expressed during the first wave of the COVID-19 outbreak, thus allowing unrestricted infection scenarios. The first epidemic wave also involved a higher global fatality rate reaching 15.2% (13). The Mexican population, with a high SARS-CoV-2 infection risk due to populated territorial clusters and high incidence of metabolic and cardiovascular chronic diseases in the world, was suitable to address this research (14–16). Previous efforts in Mexico mainly focused on demonstrating the CDs association with COVID-19 clinic course and mortality, thereby lacking a mechanistic epidemiological framework (17–20). This comprehensive study contributes to understanding the epidemiological behavior of new diseases in human populations and provides insights for surveillance and prevention of potential zoonotic outbreaks (21). Moreover, this study was based on big data associated with 509,539 official RT-qPCR test results, comprising 24.4 million metadata (22), which were putatively related to *B.1*. and *B.1.1*. SARS-CoV-2 lineages (23, 24), representing the onset-exponential phase of the first epidemic wave in Mexico (28 February to 30 June 2020). Our approach was to determine the subpopulation structure of infection in ambulatory and hospitalized cases, associated with 10 CDs and nonchronic diseases (NOCDs), considering age and sex as demographic factors in a cohort of 226,089 accumulated positive and 283,450 negative individuals, including 27,769 deaths. Therefore, the objective of this study was to establish the subpopulation attributes toward SARS-CoV-2 infection and the contribution of CDs and baseline demographic factors in shaping population vulnerability under the assumption of unrestricted immunological responses, treatments availability, and preventive constraints for contagion during the onset of the first epidemic wave.

## Materials and methods

### COVID-19 data source

**Figure 1:**
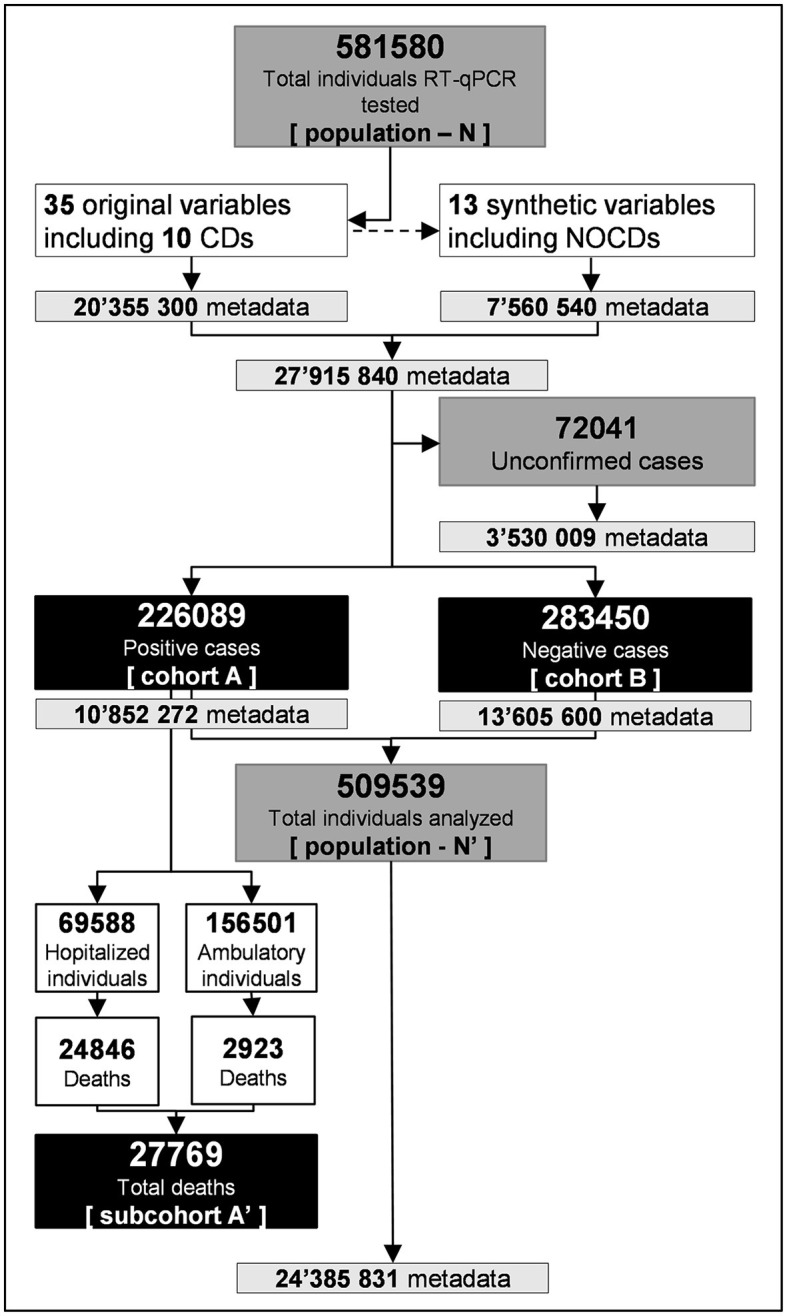
Data extraction flow (black boxes) from 581,580 official database entries accumulated during the onset-exponential phase of the first COVID-19 epidemic wave in México (population-N), from 28 February to 30 June 2020. The final big data matrix was associated with 509,539 total individuals analyzed comprising 24.4 million metadata conform in cohort A with 226,089 RT-qPCR-positive cases including subcohort A' with 27,769 deaths and cohort B with 283,450 negative cases.

The first step was to collect the official COVID-19 public databases (MS Excel^®^, dBase-COVID) of the Mexican Ministry of Health (25), from the first positive SARS-CoV-2 reported on 28 February to 30 June 2020, selected for comprising the onset-exponential phase of the first epidemic wave in Mexico. The dBase-COVID, updated daily, had 581,580 individual records (population–N) and 35 variables (20.4 million metadata), including state and municipal locations, diagnosis results, symptoms expression date, death date, sex, age, and 10 CDs, among others (Figure 1 and Supplementary Table 1). All diagnostic tests were officially regulated and conducted with certified protocols based on real-time reverse transcription-polymerase chain reaction (RT-qPCR).

### Metadata structure

The second step was to set up the database structure to conform the research objective. The dBase-COVID data were imported into RStudio^®^ v1.4.1106 – R Project^®^ v4.1.1 and performed in a workstation (HP Z1-G6. IntelCore i7 of 10^th^ generation). Data extraction was performed with *readxl, base, rattle*, and *dplyr* functions of Rstudio^®^. Sixteen numerical variables were transformed into categories, e.g., sex 1 = “female”, sex 2 = “male”, or CD 1 (presence of any chronic disease) = “yes”, CD 2 = “no”. The geo-location variables were transformed using the official nomenclature of the National Institute of Statistics and Geography (26). Additional 13 synthetic variables were created to potentially enhance the analyses, e.g., days with symptoms at testing or days from detection to death in the hospital settings. The final structured and conform database contained a cohort A of 226,089 positive individuals including 27,769 deaths, linked to 48 variables totaling 10′852272 metadata (Figure 1). A total of 72,041 unconfirmed RT-qPCR tests were excluded from the analysis (Figure 1). In this study, the infected cohort A was conform for all positive cases, symptomatic or asymptomatic at testing, including those individuals who eventually died. Death cases were considered subcohort A' of the infected cases (Figure 1).

To properly assess the age effect on infection, this variable was grouped into five categories (age_c_): < 29, 30–37, 38–46, 47–56, and >56 years. Similarly, 10 CDs were independently analyzed, as well as by categories (CD_c_) according to clinical typology: metabolic (diabetes, obesity, immunosuppressants, and chronic kidney disease); cardiovascular (hypertension and cardiovascular disease); respiratory (asthma, COPD, and smoking), even though smoking is not a CD, it was considered due to implications on pulmonary diseases; “other-CDs” (this general category was specified as such in original data matrix); and a nonchronic disease (NOCD) category for the absence of any reported CD on the dataset.

### Onset-exponential phase modeling

The third step was to confirm and characterize the onset-exponential epidemic phase intensity by fitting it to the exponential model and comparing 10 COVID-19 epidemics selected from an equal number of countries with the highest reported positive cases at the first wave onset (1). The significant epidemic rate-*r*_*e*_ estimation was fundamental to validate the fastest contagion assumption required to prove the working hypothesis. The comparison among epidemics to depict Mexico's scenario framed the study assumptions' validity. The plotting of all curves characterization was performed with *ggplot* function of RStudio^®^ using cumulative daily (*x*) positive cases from onset (*y*_*o*_) to the inflection curve point. The positive and death data (*y*) were independently fitted in SAS^®^ v9.4 using the nonlinear model: $ŷ={y_{o}}^{r_{e}\left(x\right)}$. The r_e_-parameter and *y*_*o*_ estimated the exponential epidemic rate and positive cases of primary infection, respectively. The goodness-of-fit (R^2^) and significance level (*p* < 0.0001) were obtained for comparison purposes.

### Probabilistic risk categorization for infection and mortality

The fourth step was to conduct an independent risk categorization analysis for the infection cohort A and mortality subcohort A' by using two approaches: the classification and regression tree (CART) and Spearman's *rho* correlation linked to a clustering analysis. CART allows for identifying and weighting tree-decision rules to generate splitting stratified groups of similar risk toward SARS-CoV-2. These rules were fitted using *rpart, rpart.plot*, and *prp* functions and the analysis of variance (ANOVA) among groups in RStudio^®^. The *rpart* and *rpart.plot* best-fitting function for major splitting generated an overall *complexity parameter* (*cp*) value, *cp* = 0.000003 (*p* = 0.001) and *cp* = 0.000024 (*p* = 0.001), for infection and mortality CART, respectively. The splitting stratification process runs *n-*iterations for each encountered group until a homogeneity value lower than the *complexity parameter* (*cp*) is reached, thus providing the optimal solution. This parameter estimated and compared the variance homogeneity within groups for the final decision. Each CART was fitted as multiple regression model: *y*_*i*_ = *x*_1_ + *x*_2_ + *x*_3_*... x*_*n*_, where *y*_*i*_ was the infected or death cases as dependent variables, and *x*_1_*... x*_*n*_ were 10 CDs, NOCD, age, and sex as variable predictors. Finally, with *prp*, a risk tree was built via cross-validation, thus creating stratified groups at the lowest error (27). Only nodes with statistically significant *p*-values (*p* ≤ 0.05) were plotted. Nodes per quartile of cases number were colored using a bar-scale. The CART procedure was selected because (1) it establishes rules based on multivariate criteria to explain overall variance (28); (2) it does not make any statistical distribution assumptions associated with dependent or independent variables (29, 30); and (3) it stratifies and classifies data based on weighted variables to create high- or low-risk homologous groups (30).

The second approach used was Spearman's correlation matrix based on 10 CDs, NOCD, age, and sex variables for pairings *rho* estimations. Furthermore, a hierarchical cluster analysis was performed using the Euclidian distance of *rho*-values as a dissimilarity measure among clusters and Ward's minimum variance to minimize the within-cluster variance. Independent dendrograms for the infection cohort A and mortality subcohort A' were plotted with the *tanglegram* function of RStudio^®^ for comparison purposes. In addition, per dendrogram, the infection and mortality relative risk (r) for tree clusters were estimated with *r* = *[y*_/_∑*y] 100*, where *y* is the total infected or death cases and ∑*y* is the total infected cohort A or mortality subcohort A' (Figure 1). Spearman's correlation matrix and clustering were selected because 1) it standardize data based on the variables' association level, reducing the effect of sample size and 2) it allow estimating a statistical significance (*p* ≤ 0.05).

The fifth step was to perform analogous analyses with cohort B comprising 283,450 negative cases, assuming individual exposure to the SARS-CoV-2 virus by social contact with positive cases. The purpose was to analyze the whole population–N' structure toward SARS-CoV-2 infection risk. The overall analyses included 509,539 individuals and 24.4 million metadata (population–N', Figure 1).

### A deterministic risk categorization for mortality

To further explain the implication of CD categories on COVID-19 mortality subcohort A' (Figure 1), two relative epidemiological indices were developed to estimate the mortality stratified by age_c_ and sex. A *mortality index* (MoI) was calculated with the following equation:


$$MoI=\frac{\sum_{ij}^{n}Deaths_{ij}}{\sum_{j}^{n}Cases_{j}}$$

where *Cases*_*j*_ is the number of positive individuals in *j*; *i* represents the age_c_ category from n =1 to 5; *j* is the CD_c_ category from n =1 to 4; and NOCD.

A *mortality-weighted index* (MWI), weighted by the average ($\bar{x}$) of age in each category, was calculated with the next equation:


$$MWI=\frac{{\sum_{ij}^{n}Deaths}_{ij}*{\bar{x}}_{ij}}{\sum Deaths}$$

where *i* and *j* are described as beforehand.

## Results

### SARS-CoV-2 metadata structure

**Figure 2:**
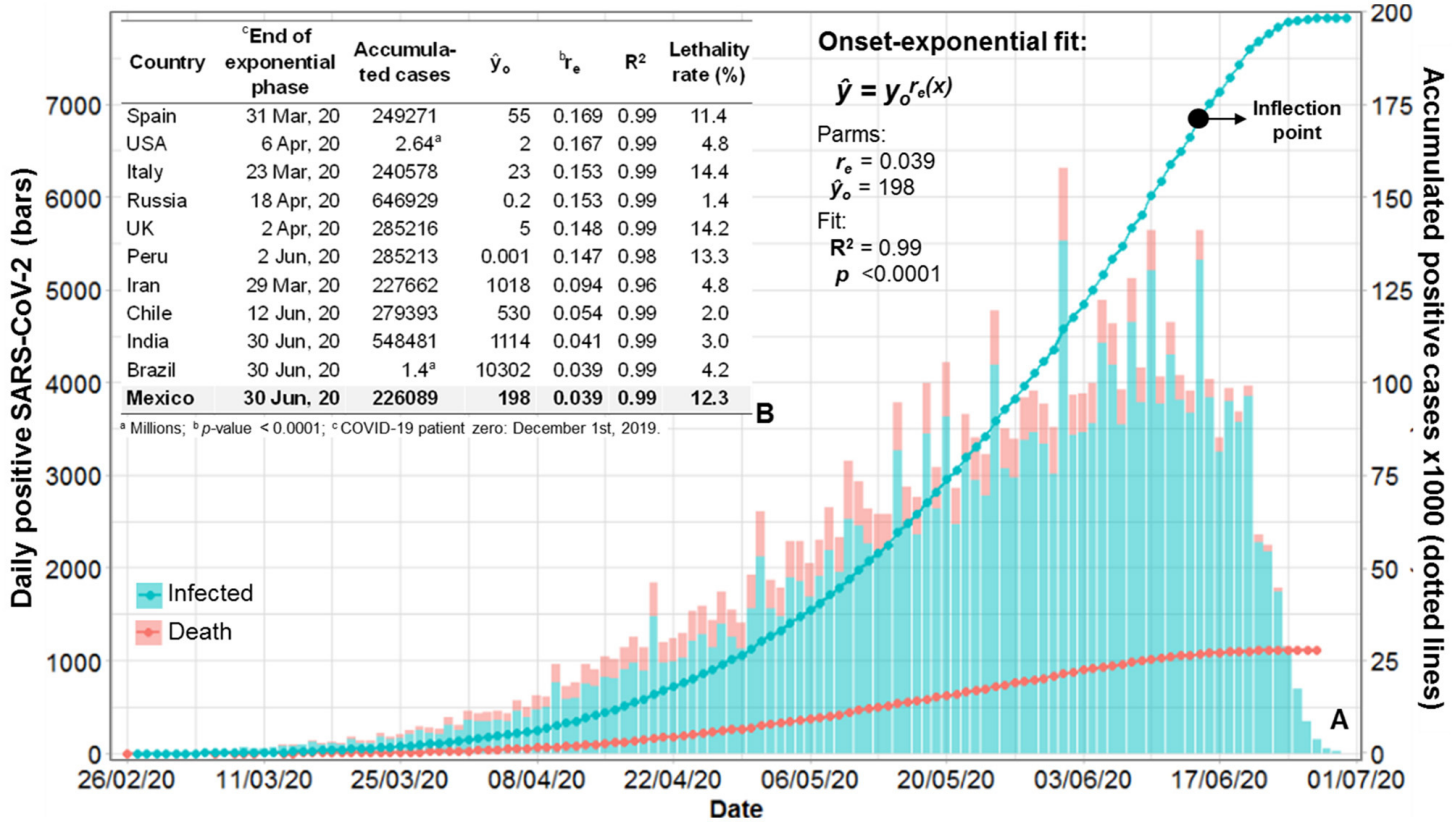
Confirmatory modeling of the first COVID-19 exponential epidemic phase in Mexico and selected countries. **(A)** First COVID-19 epidemic wave in México from 27 February to 30 June 2020. Absolute (bars) and cumulative (lines) daily cases of RT-qPCR-positive individuals representing the infected cohort A and the mortality subcohort A'. **(B)** Estimated epidemic rate (r_e_) fitted (R^2^ and *p*-value) with the exponential model from the onset (y_o_) to the curve inflection point (•) of the first COVID-19 wave in Mexico compared with that of ten countries with the highest infection accumulated positive cases. Lethality rate and epidemic rate variability (r_e_ = 0.04–0.17), with high fitting model precision (R^2^ > 0.96) indicate fast but differential SARS-CoV-2 spreading on populations. Source: Original data matrix of selected countries downloaded from Johns Hopkins University. All analyses were performed by authors.

A total of 226,089 individuals infected with SARS-CoV-2 during the onset-exponential phase of the first COVID-19 epidemic wave in Mexico were included in the study (cohort A; Figure 1 and Supplementary Table 1). The mean age was 45.7 years (range: 10–98 years), with 54.7% male cases. The overall positivity was 44.4% [(infected individuals/total individuals tested) x 100], with an official lethality rate of 12.3%. Hospitalized individuals accounted for 30.8% (62.2% of whom were men), with fatalities reaching 35.5% (65.1% of whom were men). The outpatient mortality was 1.8%. The data represented 32 Mexican states, of which 36% was associated with the metropolitan area of Mexico City and Mexico State with a combined 23.1 million habitants and a density of 6,163.3 and 760.2 residents by square kilometer, respectively. The data comprised a well-conform exponential phase as in selected comparative epidemics but with a relatively lower epidemic rate (r_e_ = 0.040 units day^1^), in contrast to Spain, USA, Italy, Russia, the UK, and Peru, which ranged from 0.15 to 0.17 (Figure 2A). In all cases, the exponential model fitted with R^2^ > 0.96 (Figure 2B). The lethality rate of Mexico was among the highest, in conjunction with Italy, Spain, UK, and Peru.

The infected cohort A, i.e., the total number of positive individuals regardless of the COVID-19 outcome, included 52.8% with NOCDs (53.9% of whom were men). The remaining 47.2% exhibited at least one CD (55.5% of whom were men), representing 27.1, 13.1, and 7% of single CD, comorbidity, and multimorbidity, respectively. The most reported chronic diseases were obesity (20,539 cases, 52.3% men) and hypertension (14,048 cases, 54.1% men). Grouped into categories, metabolic diseases (i.e., diabetes, obesity, immunosuppressants, and CKD) represented 39.7% of CD cases (Table 1). Diabetes–hypertension (4.5%) and diabetes–hypertension–obesity (2%) were the most prevalent comorbidity and multimorbidity, respectively. The control dataset (cohort B), with 283,450 RT-qPCR negative cases (Figure 1), had similar age, gender, and CD structure to cohort A.

**Table 1:** Structure of 226,089 SARS-CoV-2 infected cohort A, including the subcohort A' with 27,769 mortality cases, and MoI and MWI epidemiological relative indices adjusted by age and sex.

**Category**	**Age**	$\bar{x}$ age	**Men**			**Women**			**Total**	
			**Death**	**Cases**	**MoI** ^x^	**Death**	**Cases**	**MoI**	**Deaths/cases**	**MoI**
Metabolic	< 29	24.6	124	3,077	0.040	126	3,077	0.041	250/6,154	0.041
(diabetes, obesity, Imm, and CKD)	29–37	33.7	438	5,381	0.081	195	4,746	0.041	633/10,127	0.063
	37–46	42.3	1,243	9,001	0.138	601	7,534	0.080	1,844/16,535	0.112
	46–56	51.4	2,730	12,267	0.223	1,549	10,589	0.146	4,279/22,856	0.187
	> 56	67	7,004	18,038	0.388	5,137	16,089	0.319	12,141/34,127	0.356
**Subtotal**			**11,539**	**47,764**	**0.242**	**7,608**	**42,035**	**0.181**	**19,147/89,799**	**0.213**
**MWI** ^**Y**^			**37.22**			**48.47**			**25.36**	
Cardiovascular	< 29	23.9	44	689	0.064	46	504	0.091	90/1,193	0.075
(hypertension and CVD)	29–37	33.9	118	1,506	0.078	62	1,004	0.062	180/2,510	0.072
	37–46	42.6	504	3,569	0.141	260	2,812	0.092	764/6,381	0.120
	46–56	51.6	1,421	6,675	0.213	843	5,760	0.146	2,264/12,435	0.182
	>56	70	5,771	15,045	0.384	4,114	12,991	0.317	9,885/28,036	0.353
**Subtotal**			**7,858**	**27,484**	**0.286**	**5,325**	**23,071**	**0.231**	**13,183/50,555**	**0.261**
**MWI**			**27.49**			**36.39**			**18.85**	
Respiratory	< 29	24.4	44	2,681	0.016	16	1,663	0.010	60/4,344	0.014
(COPD, asthma, and smoking)	29–37	33.5	129	3,102	0.042	35	1,994	0.018	164/5,096	0.032
	37–46	41.6	312	3,292	0.095	95	2,204	0.043	407/5,496	0.074
	46–56	51	569	3,194	0.178	223	2,071	0.108	792/5,265	0.150
	>56	68	2,224	5,705	0.390	976	3,117	0.313	3,200/8,822	0.363
**Subtotal**			**3,278**	**17,974**	**0.182**	**1,345**	**11,049**	**0.122**	**4,623/29,023**	**0.159**
**MWI**			**10.88**			**8.80**			**6.28**	
Other CDs	< 29	20.7	16	207	0.077	10	260	0.038	26/467	0.056
	29–37	33.7	7	176	0.040	2	308	0.006	9/484	0.019
	37–46	42	19	224	0.085	19	328	0.058	38/552	0.069
	46–56	51.1	36	189	0.190	23	276	0.083	59/465	0.127
	>56	68	128	276	0.464	60	220	0.273	188/496	0.379
**Subtotal**			**206**	**1,072**	**0.192**	**114**	**1,392**	**0.082**	**320/2,464**	**0.130**
**MWI**			**0.65**			**0.67**			**0.41**	
Nonchronic disease	< 29	22.9	117	13,554	0.009	58	14,070	0.004	175/27,624	0.006
	29–37	33.5	275	13,678	0.020	96	12,917	0.007	371/26,595	0.014
	37–46	41.9	721	14,262	0.051	231	12,136	0.019	952/26,398	0.036
	46–56	51.1	1,468	11,960	0.123	426	9,262	0.046	1,894/21,222	0.089
	>56	66	3,058	10,948	0.279	1,242	6,649	0.187	4,300/17,597	0.244
**Subtotal**			**5,639**	**64,402**	**0.088**	**2,053**	**55,034**	**0.037**	**7,692/119,436**	**0.064**
**MWI**			**17.50**			**12.50**			**9.75**	
**Total**			**18,289**	**123,616**		**9,480**	**102,473**		**27,769/226,089**	

COVID-19 data of the first onset-exponential epidemic phase in Mexico.

^*X*^Relative mortality index (MoI) associated to CDc and age_c_. ^*Y*^Relative mortality-weighted index associated to CDc (MWI).

### Probabilistic risk categorization for SARS-CoV-2 infection and mortality

**Figure 3:**
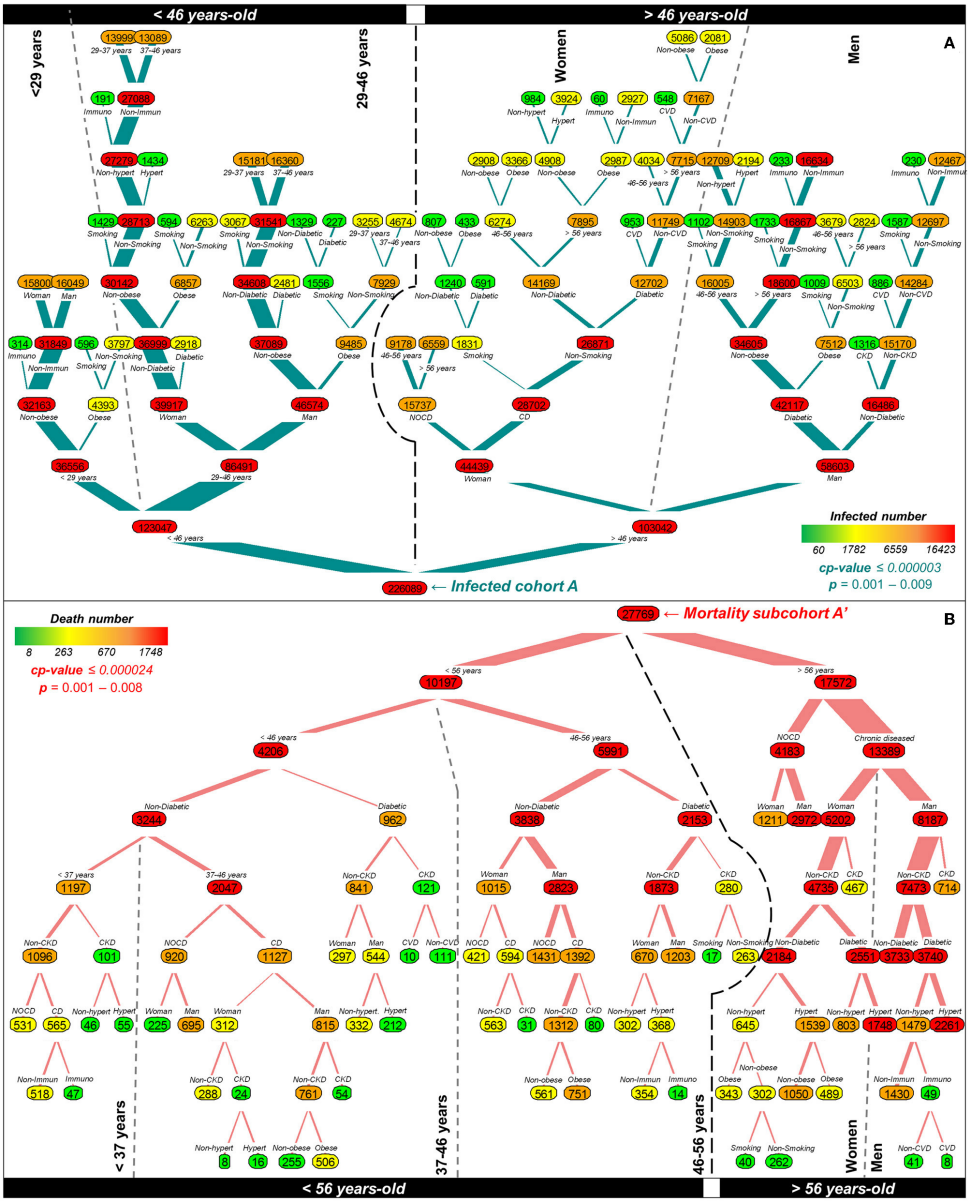
Tree risk categorization of infection and mortality due to SARS-CoV-2 during the onset-exponential phase of the first COVID-19 epidemic wave in Mexico based on 226,089 positive cases and 10′857,272 metadata records comprising 13 variables including NOCD and CDs. Branch thickness represents the main root of significant risk. The colored bar scale represents the number range of positive cases applied to nodes. **(A)** The major virus infection risk implicated four main branches, highlighted by upper black boxes and vertical dotted lines, determined primarily by age, followed by sex, with a *cp*-value ≤ 0.000003 (*p* < 0.009). The infection risk for individuals with NOCD represented 52.5%. **(B)** The mortality risk was also influenced by age (*cp-*value ≤ 0.000024; *p* < 0.008) but conditioned by chronic diseases with a higher association in older to 56 years (48%) and splitting the risk by sex (women = 38.9%; men = 61.1%). NOCD accounted for 26.9% of the mortality risk. Main and secondary tree branches are highlighted in bottom black boxes and vertical dotted lines, respectively.

As the first probabilistic classificatory approach applied to the infected cohort A (226,089 individuals), tree risk categorization significantly selected age as the primary factor of infection risk, with 46.9% of the explained variance (*cp*-value = 0.000003) (Figure 3A). The age cutoff onto two main probabilistic branches, from which a significant classificatory risk node was derived, was 46 years, which represented 123,047 (*p* = 0.001–0.009) and 103,042 (*p* = 0.001–0.002) for younger and older than the significant age cutoff, respectively. Furthermore, age (29 years) and sex were the second most significant subordinated factors toward infection (*p* = 0.001). Notably, NOCD represented only 6.4% of the explained variance due to restricted probabilistic combinations only within age and sex toward infection. This restricted determination resulted in 58,679 and 28,204 infection cases associated with NOCD in the root branches determined by sex and age (29–46 years), respectively.

A similar low variance contribution was found on infection associated with CDs. The type of disease determined the probability of infection in individuals with one CD, conditioned by age, being higher in those individuals exhibiting diabetes (12.3%, *cp* = 0.0006), hypertension (10.1%, *cp* = 0.0001), and obesity (7.8%, *cp* = 0.001) accounting for a total of 30.2%. Sex contributed 9.7% of the overall infection risk, mainly associated with ages older than 46 years. For instance, for women younger and older than 46 years threshold, 12,829 and 28,702 had SARS-CoV-2 infection, respectively, and exhibited at least one chronic disease (Figure 3A). For the same contrasting risk scenario considering only diabetic women, there were 2,918 and 13,293 positive cases for younger and older than the 46-year cutoff, respectively. Notably, this combinatory effect was even higher in men, with 2,481 and 42,117 cases, indicating a higher infection probability in diabetic older men than in diabetic women. In individuals younger than 29 years, the infection risk associated with those exhibiting at least one CD was 25.6%. The remaining CDs cases, independent of sex, were associated with obesity (4,393 cases), smoking (596 cases), and immunosuppressants (314 cases) with a risk of 14.5%. Other CDs, such as kidney (CKD), cardiovascular (CVD), smoking, and immunosuppressants, accounted for 6.9% of the infection risk variance. Lower risk of infection, but significant (*p* = 0.009), probably due to underrepresentation in cohort A, was found to be associated with individuals with comorbidities, i.e., more than one CD, such as obesity and smoking (1,556 men); diabetes–obesity–smoking (1,009 men); diabetes–CVD (953 women); and obesity–immunosuppressant (60 women) (Figure 3A).

In the restricted analyses of the mortality subcohort A' (27,769 cases), age was again the primary significant risk factor with 72.3% of the explained variance conditioned by the type of CD (*cp*-value < 0.000024), from which a significant tree classification risk was derived upon a 56-year cutoff (*p* = 0.001–0.008) (Figure 3B). NOCD accounted only for 3.7% of the variance, representing 26.9% of all death cases (*cp* = 0.0006, *p* = 0.001). A robust significant risk, representing 48% of the cases, was composed of women and men (w, m) older than the 56-year cutoff who mainly exhibited diabetes (19% women, 27.9% men; *cp* = 0.0004), hypertension (11.5% women, nonsignificant in men, *cp* = 0.0001), and CKD (3.5% women, 5.3% men; *cp* = 0.0009). Deaths with comorbidity combinations involving diabetes were significantly associated with CKD among patients aged 56 years, regardless of sex (401 cases). Conversely, in those individuals older than 56 years, diabetes was significantly combined with hypertension (1,748 women and 2,261 men) (Figure 3B). Multimorbidity disease significant combinations, regardless of the age category, included diabetes–CKD–CVD (10 cases), diabetes–hypertension–immunosuppressants (14 women), and diabetes–hypertension–CVD (8 men) (Figure 3B). For individuals younger than 29 years, mortality was independent of sex and mainly associated with obesity and hypertension (60 and 32 deaths, respectively). However, this node was not significant.

**Figure 4:**
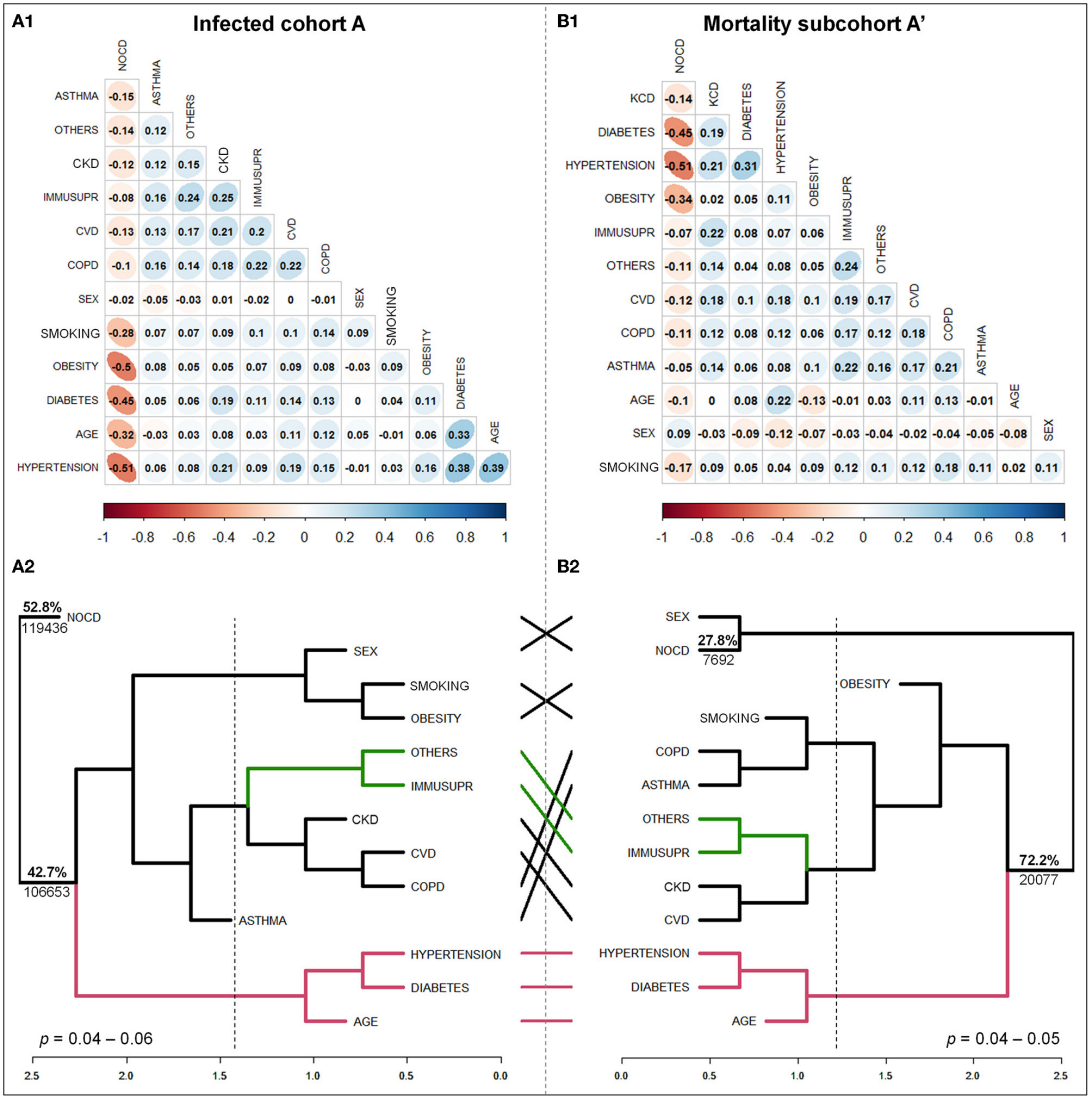
Differential risk structure toward infection **(A1, A2)** and mortality **(B1, B2)** due to SARS-CoV-2 based on Spearman's *rho*-values estimated with 13 variables, comprising sex, age, nonchronic (NOCD), and 10 chronic (CDs) non-infectious diseases associated with 226,089 infected individuals during the onset-exponential phase of the first COVID-19 epidemic wave in Mexico. **(A1, B1)**: correlation matrix for the infected cohort A and mortality subcohort A', respectively. The colored bar-scale represents the *rho*-value. If closer to ± 1 indicates a higher correlation between variables. **(A2, B2)**: Dendrogram of rho, linked to cluster analyses for the infected cohort A and mortality subcohort A', shows respectively, a clear independent and dependent risk effect on CD, age, and sex, respectively. The scale at the bottom represents the dissimilarity of Euclidean distance. The dotted line represents the cutoff for risk-cluster conformation, and the percentage is the estimated risk based on positive cases associated with a specific branch (*p* = 0.04–0.06). Lines connecting dendrograms identify the clustering variables. Others. Other CDs.

The second probabilistic associative approach applied to the infected cohort A and subcohort A', based on the matrix of the Spearman's *rho*-values (Figures 4A1, B1), confirmed that CD, age, and sex did not fully explain infection risk toward SARS-CoV-2. NOCD, with 52.8% of infection probability and conform for 119,436 positive cases, represented a well-separated independent cluster (*p* = 0.05) at a Euclidean distance of 1.4 cutoff. The CDs and demographic factors formed four risk clusters with 47.2% infection probability (Figure 4A2) (*p* = 0.04–0.06). Age and sex conform a cluster with diabetes and hypertension, and obesity and smoking, respectively (*p* = 0.05). The infection risk increased to 25.4% and 15.3%, respectively, for individuals who presented the two diseases (i.e., comorbidity). Age influenced the vulnerability of older people with diabetes or hypertension to developing SARS-CoV-2 infection (*p* < 0.00001). Age-related associations with diabetes and hypertension had the highest positive *rho*-values of 0.39 and 0.33, respectively (Figure 4A1).

Contrary to the infection scenario and targeting only the subcohort, the higher probability for mortality was associated with CDs and age totaling 72.2% (Figure 4B2) (*p* = 0.05; rho = −0.51–0.31). NOCD and sex defined a well-distant risk cluster of 27.8% (*p* = 0.05). Older people with comorbidity of diabetes–hypertension (*p* = 0.04) had an increased risk of death at 60.5%, whereas those with a single CD accounted for only 18.8%. These conditions were more determinant over the threshold of 56 years (Figure 4B2). The comparison between infection and mortality dendrograms showed a slight displacement of risk-cluster location with an estimated 66% similarity, thus indicating differences in influencing health factors toward SARS-CoV-2 outcome (Figures 4A2, B2).

In the negative cases of cohort B, the variance structure was similar to cohort A (Supplementary Figures 1A, 2 and Supplementary Table 2). The primary statistically significant age cutoff was also 46 years (*p* = 0.001–0.007) (Supplementary Figure 1A). For those older than 46 years (51,206), smokers were the first cutoff linked to obesity. Meanwhile, nonsmokers were associated with diabetes, obesity, and hypertension. For those under 46 years (58,337), obesity was the leading cutoff, but linked to diabetes and smokers. In NOCD-negative individuals (173,907), the population structure variance was determined only by sex and age as expected (Supplementary Figure 1 and Supplementary Table 2). The cluster structure was also similar to positive SARS-CoV-2 in cases of cohort A. The cross-dendrogram correlation revealed associativity of r^2^ = 0.93 among cohorts. Notably, asthma was included in the sex–obesity–smoking cluster (Supplementary Figure 2).

### SARS-CoV-2 relative *mortality indices*

**Figure 5:**
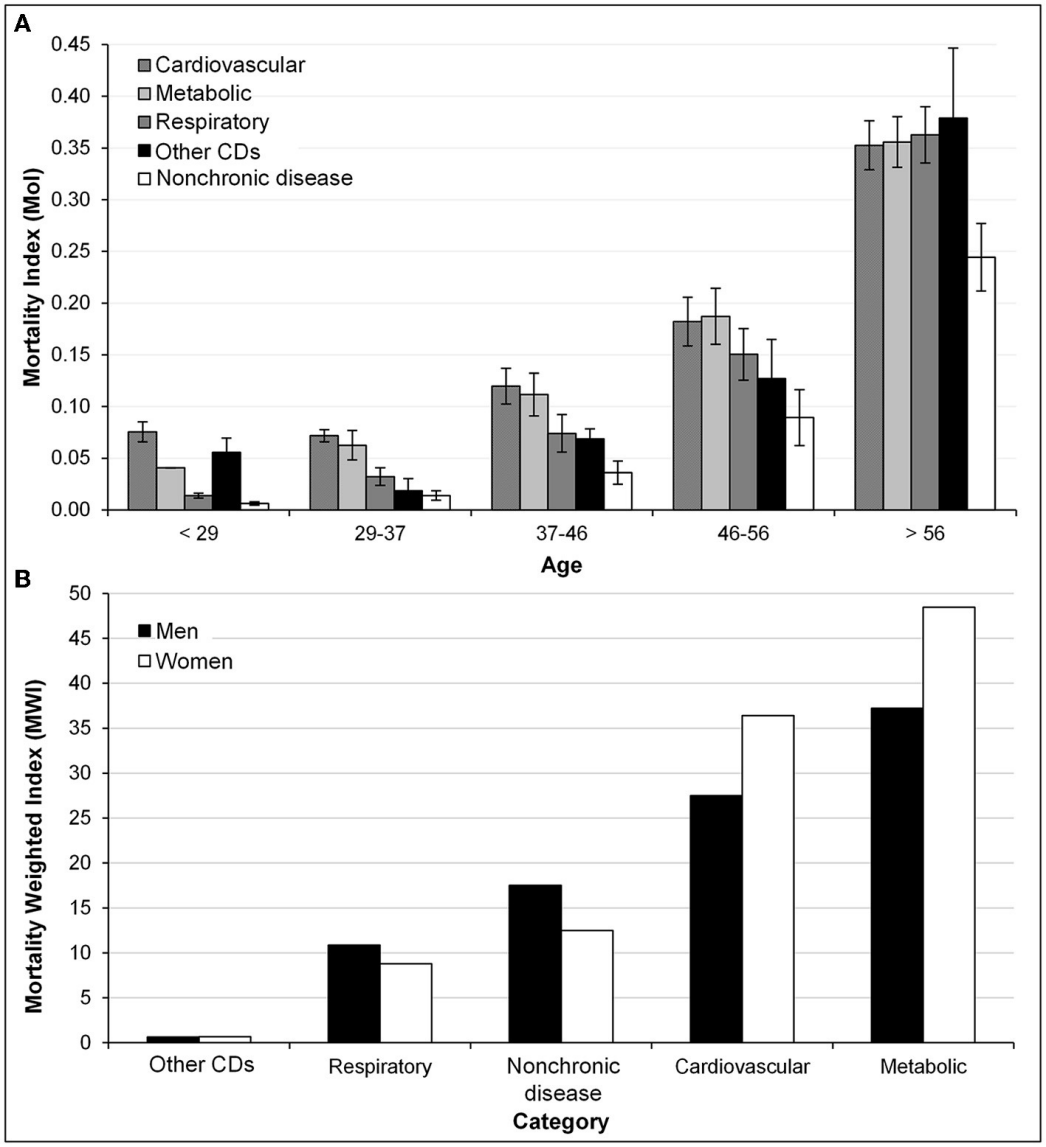
Association of SARS-CoV-2 positive individuals exhibiting nonchronic diseases (NOCD) or any CD within five categorized chronic diseases (CD_c_) with COVID-19 mortality at the first exponential epidemic phase in Mexico. **(A)** Differential increase of relative *mortality index* (MoI) values on individuals with NOCD and CD_c_ upon age category increase (age_c_). Bars represent the standard deviation. **(B)** Differential effect on relative mortality-weighted index (MWI) values of men versus women on respiratory and NOCD (higher) and on cardiovascular and metabolic category (lower).

The relative *mortality index* (MoI) stratified by age confirmed the differential effect of CD category (CD_c_) and NOCD on mortality (Figure 5A). Cardiovascular and metabolic diseases represented the higher index with 0.26 and 0.21, respectively, whereas NOCD was the lowest with 0.06 (Table 1). MoI values increased by age category (age_c_) and were higher, but similar, for patients older than 56 years among CD_c_ (0.35–0.37) compared to NOCD (0.24), thus indicating a significant conditional age effect on mortality (Figure 5A). Conversely, for ages less than 56 years, the MoI did not exhibit clear differences between CD_c_ and NOCD. As for sex, the MoI was consistently higher among men than women, independent of age, CD_c_, or NOCD (Table 1).

The relative *mortality-weighted index* (MWI) showed that mortality was also influenced by CD_c_ and sex (Figure 5B). Again, the individuals with metabolic or cardiovascular diseases were associated with a higher mortality risk index of 25.4 and 18.9, respectively, compared to NOCD (9.8). However, contrary to MoI, women were notably the most vulnerable in metabolic and cardiovascular categories with 48.5 and 37.3, respectively. Furthermore, men had a higher risk associated with respiratory diseases and NOCD (Table 1; Figure 5B). These indices were calculated relative to each CD_c_ to avoid biases due to sample underrepresentation of specific chronic disease category in cohort A.

## Discussion

Despite massive vaccination and lethality reduction, the recent COVID-19 pandemic, which was characterized by fast virus contagion, a dynamic prevalence of variants, and a reduction of the age threshold for infection, raises questions about our mechanistic comprehension of SARS-CoV-2 epidemiology at the communitarian level (6). Most studies continue to focus on an understanding of the infection clinical outcome, particularly the post-COVID condition, the development of cure treatments, and the enhancement of vaccines to include children (7, 8, 11, 31–33). However, there is still a strong need for comprehensive studies associated with virus behavior at the ambulatory population level for surveillance and prevention purposes (21). Current forecasting relies on limited clinical and hospital settings data (34–38). Moreover, current data availability and quality of detection and monitoring have been strongly compromised based on the worldwide expectation of immunization coverage to cope with the disease. The recent endemic and seasonal statement may even more discourage keeping epidemiological studies at the communitarian level (39).

This study deals with a fundamental epidemiological assumption of the preexistence of a susceptible population as a driving force for SARS-CoV-2 epidemics. Our findings challenge the presence of such a subpopulation. The analyses of 226,089 positive individuals and 10′852272 metadata records representing the specific onset-exponential first wave in Mexico (Figure 1) suggest that infection at the communitarian level relies more on infectious sources in the proximity of individuals independently of their health conditions, sex, or age as has been commonly implied (40–42). Rather than ‘choosing' vulnerable subpopulation(s), this random infection was supported by the fact that baseline chronic diseases, extensively associated with COVID-19, did not condition infection. In one probability scenario, our structural risk analyses showed that individuals with NOCD have a slightly higher infection probability (52.8%) than those exhibiting any CD, including comorbidities, without age and sex influence (*p* = 0.05). In a second scenario, a cutoff of 46-year individuals was conditioned to diverse risk categories of virus infection (*p* = 0.001–0.009). However, although age and sex have been extensively associated with COVID-19 severity and always associated with CDs under our analytical scenarios, age standalone was a significant factor in shaping the infection risk structure in the population but decreased the age threshold with respect to most reports, wherein older people appear to be more vulnerable. In such reports, the focus on the clinical evolution of inpatients may explain this discrepancy (11, 33, 38, 40, 41, 43). The independent effect of infection regarding CDs toward COVID-19 was supported by the health structure of negative cases with a similar tendency but a higher proportion of NOCDs (61.4%, *p* < 0.007) (Supplementary Figures 1, 2 and Supplementary Table 2).

After restricting the analysis to the mortality subcohort, the results are in agreement with extensive studies suggesting that CD, age, and sex are implicated in COVID-19 severity (33, 40, 41, 44–47). Nonetheless, severity is the outcome of the pathogenesis process beyond infection. This subpopulation included 89.5% of inpatients (age: 24–98 years; men and women 1.9:1). However, in our findings, CD risk categories were conditioned explicitly by age, with an age threshold of 56 years (*p* = 0.001–0.008). Moreover, an age cutoff at 46 years associated with sex was determined as a second significant risk level with some chronic diseases. Similar to other reports, mortality associated with CD increased with age (40, 44), and individuals with hypertension and diabetes, adjusted by their implication on comorbidities, had a differential increase in infection and mortality risk (44, 45, 48, 49). Moreover, sex implication for CD and COVID-19 fatality outcomes are recognized, but not a clear-cut specific association (11, 41, 49). In our results, women exhibited a higher vulnerability to death associated with metabolic diseases (i.e., diabetes, obesity, immunosuppressant, and CKD). In contrast, men showed a higher vulnerability to respiratory diseases (i.e., COPD, asthma, and smoking), even though infection in diabetic individuals was more than 3-fold concerning women.

The CD factor in our research framework was based on the suitability of the Mexican population due to the high incidence of metabolic and cardiovascular chronic diseases (14, 15). However, the analyzed metadata (*N* = 581,580) accounted for 16.2, 12.5, and 16.3% of obesity, diabetes, and hypertension, respectively, which is in contrast with the 40.2, 10.6, and 13.4% of the last official survey specifically designed to estimate the status of CD (*n* = 120,843) (50). When conceding that the slightly lower prevalence of diabetes and hypertension, and higher prevalence of obesity in the official data were the proper estimations, such values may not change our fundamental findings. Specifically, the independent SARS-CoV-2 infection probability and age are significant factors in shaping the infection risk.

These findings shape the classical paradigm of the preexistence of a specific susceptible population for the occurrence of epidemics. This may be true for diseases framed by long host–pathogen coevolutive processes and endemicity but not for pathogens encountering a new host. The SARS-CoV-2 strain diversity and mutational patterns through time and space (51, 52), as well as the parasitic fitness switch from aggressivity to spreading survival, appear to be indicators of an early evolutionary process involving a pathogen obligated to survive on the host (53). In this development, vaccination as a massive host intervention has played a minor role in comparison to host genetics and health attributes of the population itself, as inferred from this study and many clinical studies (8, 9, 51, 54, 55).

The spread of SARS-CoV-2 and pathogenicity support the rationality of these findings. The airborne virus spreading, which is the main contagious mechanism through respiratory droplets and, to a lesser extent, via aerosols (56, 57), is not host-target specific, which allows the virus acquisition by any individual upon inoculum exposure (58). Primary infection requires upper respiratory tissues for rapid multiplication before host internalization (59–61). This pathway is mediated through high angiotensin-converting enzyme 2 (ACE2) receptor expression in epithelial cells lining salivary gland ducts (60, 62), and other respiratory tissues, heart, and gastrointestinal tracts but with lower expression and infectivity (8, 55, 63). The coding gene of ACE2 is constitutive to the human genome with low protein-coding variability and no differential expression due to sex, age, or population (55, 64, 65).

Therefore, we postulate that infection with SARS-CoV-2 originates from random virus exposures rather than a specific health condition. Infection is the first stage of pathogenicity involving virus–host recognition and entry into epithelial cells to initiate virus multiplication (61). Infection may not lead to disease, as asymptomatic conditions imply (21). This scenario departs from the general usage of infection as equivalent to disease or severity [e.g., (51)]. Once the virus infection is established, health, genetics, and other determinants may play a role in the COVID-19 outcome, including asymptomatic and severe courses with acute respiratory distress syndrome, multiorgan involvement, and death (9). However, at least at the early virus replication stage, it follows an evolutionarily conserved path common to viruses, thus allowing for unrestricted multiplication (61). Current epigenetic studies have shown that ACE2 hypomethylation in the nasal epithelium can lead to increased SARS-CoV-2 infectivity and COVID-19 severity via a greater abundance of ACE2 receptors (7, 8). A meta-analysis of plasma ACE2 also demonstrated that elevated ACE2 levels had a causal relationship with COVID-19 infection, severity, and hospitalization and that a solid X-linked locus associated with ACE2 may explain sex differences in ACE2 expression across various tissues (51).

Although the framework of this extensive study was the high occurrence of obesity/overweight (33–60%), hypertension (32–45%), and diabetes (3.1–10.6%) in the Mexican population (14, 15, 50), as well as one of the highest lethality rates (12.3%), further epidemiological studies may be needed to unveil the driving question of this research. The inclusion of diverse core populations, as implied by contrasting fatalities and epidemic rates of selected countries in this study (Figure 2), may provide advanced insights when considering ethnicity and geographical disparities, coupled with significant genomic data and health determinants. However, these results encourage the imperative need for communitarian approaches to develop preventive surveillance systems. The development of algorithms to address ambulatory populations may improve COVID-19 management and cope with zoonotic threats, without assuming a specific susceptible subpopulation that is reached through clinical or hospital settings (21). Our results may also support the benefit of massive ambulatory SARS-CoV-2 testing conducted for several countries during the critical contagious stage (58), rather than using digital risk assessment or directing tests on individuals upon presumptive COVID-19 symptoms to assist disease control treatment (66–70). It is well known that asymptomatic individuals, estimated at 22.1% under lockdown conditions (58), may exhibit a comparable virus titer to those with symptoms and thus could play a significant role in transmission chains (21). A web-app surveillance platform, linked to testing at clustering labor, social, and household environments, may overcome the cost-time factors of massive testing and effectively accomplish the confinement strategy and clinical monitoring at the community level (21). Although WHO and many countries have recently declared the end of COVID-19 as public health emergency (2), the risk of new variants and emerging diseases should encourage us to continue our comprehension of this epidemic to enhance local and global preventive health systems.

## Conclusion

Based on 24.4 million metadata records associated with 509,539 official RT-qPCR cases accumulated during the onset-exponential phase of the first epidemic wave in Mexico, we provided robust epidemiological evidence to support our hypothesis that SARS-CoV-2, a novel pathogen to the human population, did not encounter a susceptible subpopulation with a specific set of health condition for the infection establishment and epidemic development. However, the clinical evolution of COVID-19, such as disease severity and mortality, was associated with vulnerability factors explicitly conditioned by age and sex, as has been extensively published. The differentiation of infection, as the process of the successful virus, entering and early multiplication in the host, independent of the disease outcome, was fundamental in this research to primarily account for an ambulatory and hospitalized cohort. The specific selection of the onset-exponential phase of the first epidemic wave was also essential to assess the cohort risk structure based on the assumptions of random population exposure to the virus due to the fast spreading of the virus (lethality rate = 12.3%, *R*_*o*_ > 1), limited and unsteady immunological response, pathogen capabilities to evade or subvert host defense mechanisms, constrained clinical knowledge for treatment, and unprepared health systems. These findings encourage the addressing of communitarian approaches to develop preventive surveillance systems to target ambulatory populations. Such systems may complement conventional and specific surveillance platforms, such as SUIVE (https://sinave.gob.mx/) or SISVER (https://sisver.sinave.gob.mx/influenza/), respectively, that are currently in operation in Mexico. This view may effectively intervene in COVID-19, which remains a global health risk, and potential zoonotic threat without assuming a specific susceptible subpopulation targeted by new pathogens with no signals at the human coevolutive microbiological core. To our knowledge, this is the first work addressing this fundamental epidemiological question.

## Limitations

The limitation of this research was derived from SARS-CoV-2 diagnostic data upon presumptive COVID-19 symptoms or associations with infected individuals. Therefore, the database does not represent an entirely random sampling of the ambulatory population. Despite the high lethality rate observed during the addressed epidemic phase, the epidemic rate was lower compared to many countries, thus restricting the sampling size and health structure of the studied population. Data on social, behavioral, and environmental determinants and cases with asymptomatic conditions were unavailable. Although confinement was not mandatory in Mexico, restricted activities limited the children and young people's movements, thus preventing data of these cohorts despite reports of less susceptibility (58).

## Data availability statement

The original contributions presented in the study are included in the article/Supplementary material, further inquiries can be directed to the corresponding author.

## Author contributions

GM-A was responsible for the conception, conceptualization, and design of the study. JC-C and GA-S were responsible for the data acquisition. GA-S and GM-A were responsible for the statistical analyses. GM-A, GA-S, and IÁ-M were responsible for the preparation of the manuscript. All authors contributed to the article and approved the submitted version.
