# Supplementary material for: Were metabolic and other chronic diseases the driven onset epidemic forces of COVID-19 in Mexico?
Source: Front Public Health. 2023 Aug 7;11:995602. doi: 10.3389/fpubh.2023.995602 (PMC10441236; doi:10.3389/fpubh.2023.995602)
Supplement: Supplementary file 1 [file Table_1.DOCX]

**Supplementary materials**

**Table S1**. Description and characteristics of variables included the official dBase-COVID used for data extraction and conformation of analytical data matrix (see Figure 1).

| ***No.*** | ***Variable name*** | ***Type variable*** | ***Response variable*** | ***Levels*** |
| --- | --- | --- | --- | --- |
| **1** | Date actualization | Date | Date | dd-mm-yyyy |
| **2** | Id record | Id | Id | n… |
| **3** | Origen | Categorical | In/out USMER | 2 |
| **4** | Sector | Categorical | Type hospital | 14 |
| **5** | State | Categorical | Name states | 32 |
| **6** | Sex | Categorical | Male / Female | 2 |
| **7** | State born | Categorical | Name states | 32 |
| **8** | State residence | Categorical | Name states | 32 |
| **9** | County residence | Categorical | Name county | 2500 |
| **10** | Type patient | Binary | Hospitalized / Ambulatory | 2 |
| **11** | Date register | Date | Date | dd-mm-yyyy |
| **12** | Date initial symptoms | Date | Date | dd-mm-yyyy |
| **13** | Date death | Date | Date | dd-mm-yyyy |
| **14** | Patient intubated | Binary | Yes or No | 2 |
| **15** | Patient with pneumonia | Binary | Yes or No | 2 |
| **16** | Age | Continuous | Number | n… |
| **17** | Nationality | Categorical | Country | n… |
| **18** | Pregnancy | Binary | Yes or No | 2 |
| **19** | Speak native language | Binary | Yes or No | 2 |
| **20** | Diabetes | Binary | Yes or No | 2 |
| **21** | COPD | Binary | Yes or No | 2 |
| **22** | Asthma | Binary | Yes or No | 2 |
| **23** | Immunosuppressive | Binary | Yes or No | 2 |
| **24** | Hypertension | Binary | Yes or No | 2 |
| **25** | Other comorbidities | Binary | Yes or No | 2 |
| **26** | Cardiovascular (CVD) | Binary | Yes or No | 2 |
| **27** | Obesity | Binary | Yes or No | 2 |
| **28** | Chronic renal (CKD) | Binary | Yes or No | 2 |
| **29** | Smoke | Binary | Yes or No | 2 |
| **30** | Other comorbidity | Binary | Yes or No | 2 |
| **31** | Diagnostic SARS-COV-2 | Categorical | Positive, Negative or Pending | 2 |
| **32** | Migrant | Binary | Yes or No | 2 |
| **33** | Nationality | Categorical | National or Foreign | 2 |
| **34** | Origen country | Categorical | Country | n… |
| **35** | ICU (Intensive Care Unit) | Binary | Yes or No | 2 |


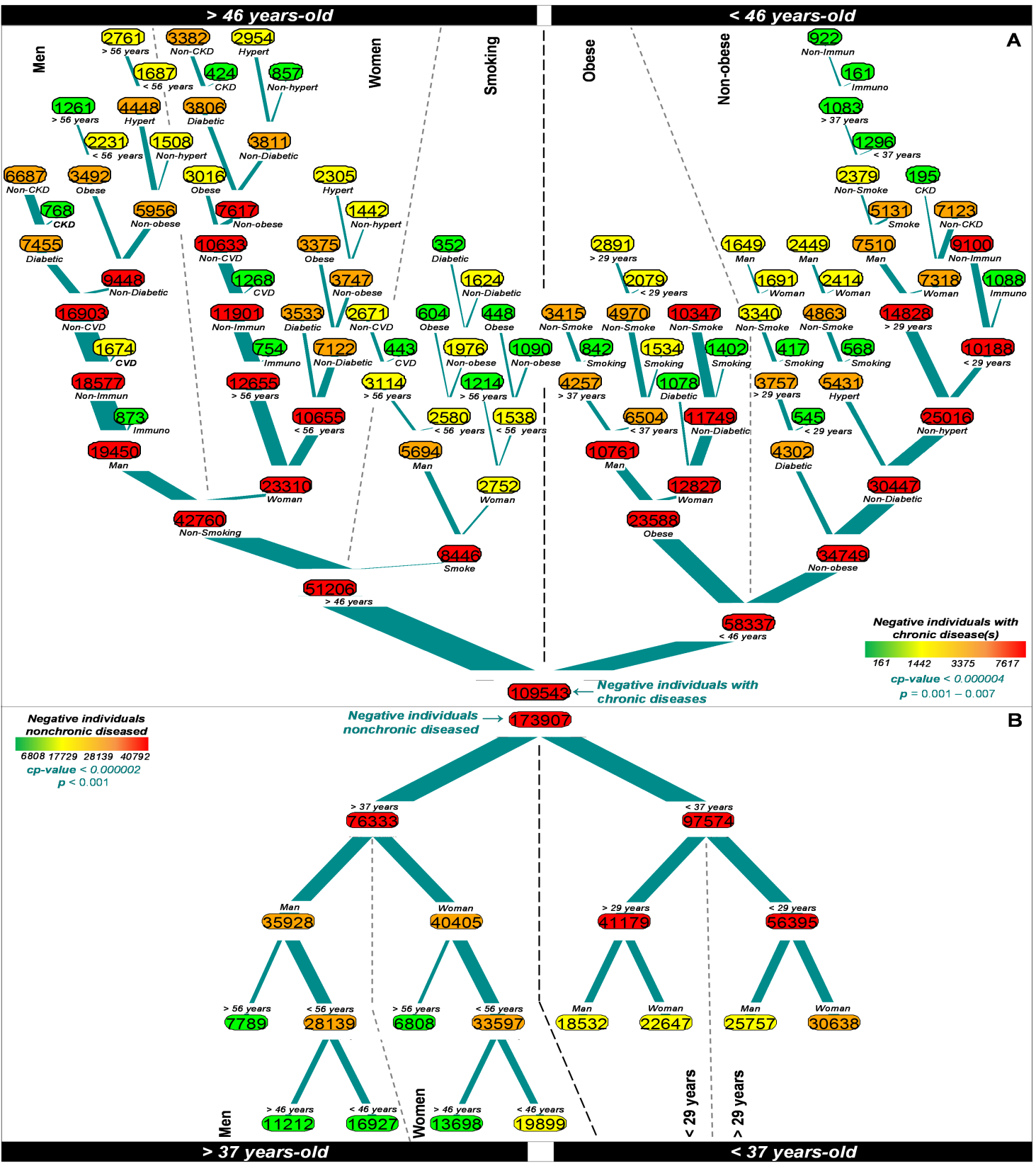


**Figure S1.** Tree risk categorization of negative cohort B during the onset-exponential phase of the first COVID-19 epidemic wave in Mexico based on 283450 negative cases and 13’605,600 metadata records comprising 13 variables including NOCD and CDs. Branch thickness represents the main root of significant risk. The colored bar-scale represents the number range of positive cases applied to nodes. **A)** The negative individuals with chronic diseases structure implicated four main branches, highlighted by upper black boxes and vertical dotted lines, determined primarily by age, followed by smoking, and obesity with a *cp*-value ≤ 0.000004 (*p <* 0.007). **B)** The negative individuals of nonchronic diseased structure influenced by age and sex (*cp-*value ≤ 0.0000002; *p* < 0.001). Main and secondary tree branches are highlighted in bottom black boxes and vertical dotted lines, respectively.


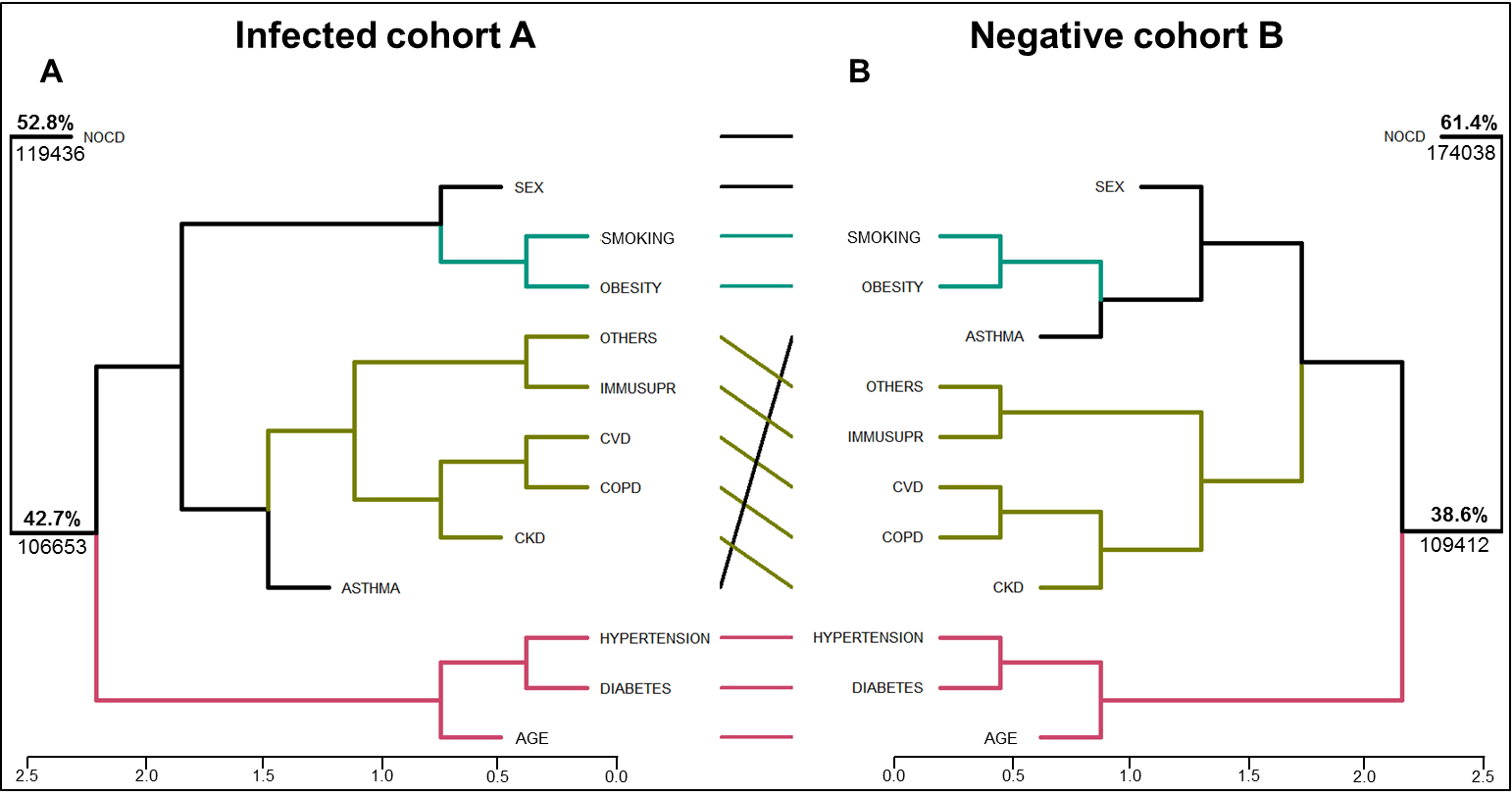


**Figure S2.** Differential risk structure for SARS-CoV-2 infection cohort A (**A**) and negative cohort B (**B**) based on Spearman´s *rho*-values estimated with 13 variables, comprising sex, age, nonchronic (NOCD), and 10 chronic (CDs) non-infectious diseases associated to 226089 infected individuals and 283450 negative cases, respectively, during the onset-exponential phase of the first COVID-19 epidemic wave in Mexico. Dendrograms of *rho*-values showing similar structure (r^2^ = 0.93) on CD, NOCD, age and sex.

**Table S2.** Structure of 283450 negative cohort B, including the subcohort, including 7158 fatality cases, by age and sex cases. COVID-19 data of the first onset-exponential epidemic phase in Mexico.

|  |  |  | **Man** | | | **Woman** | | | **Total** | | |
| --- | --- | --- | --- | --- | --- | --- | --- | --- | --- | --- | --- |
|  | **Age** | $\bar{\boldsymbol{x}}$  **age** | **Deaths** | **Cases** | **Fatal.** | **Deaths** | **Cases** | **Fatal.** | **Deaths** | **Cases** | **Fatal.** |
| **Metabolic**  (Diabetes, Obesity, Imm, and CKD) | < 29 | 18.8 | 81 | 3276 | 0.024 | 71 | 3465 | 0.020 | 152 | 6741 | 0.023 |
|  | 29 - 37 | 32.9 | 88 | 4479 | 0.019 | 54 | 4997 | 0.011 | 142 | 9476 | 0.015 |
|  | 37 - 46 | 41.4 | 186 | 5748 | 0.031 | 92 | 6973 | 0.013 | 278 | 12721 | 0.022 |
|  | 46 - 56 | 50.4 | 424 | 6334 | 0.063 | 251 | 7968 | 0.031 | 675 | 14302 | 0.047 |
|  | > 56 | 69.1 | 1458 | 8173 | 0.151 | 1144 | 8768 | 0.115 | 2602 | 16941 | 0.154 |
| **Subtotal** |  |  | **2237** | **30247** | **0.069** | **1612** | **33783** | **0.046** | **3849** | **64030** | **0.060** |
| **Cardiovascular**  (Hypertension and CVD) | < 29 | 22.0 | 19 | 491 | 0.037 | 13 | 452 | 0.028 | 32 | 943 | 0.034 |
|  | 29 - 37 | 32.6 | 6 | 795 | 0.007 | 6 | 702 | 0.009 | 12 | 1497 | 0.008 |
|  | 37 - 46 | 41.1 | 22 | 1472 | 0.015 | 12 | 1496 | 0.008 | 34 | 2968 | 0.012 |
|  | 46 - 56 | 50.3 | 78 | 1980 | 0.038 | 32 | 2457 | 0.013 | 110 | 4437 | 0.025 |
|  | > 56 | 67.0 | 434 | 3443 | 0.112 | 309 | 3372 | 0.083 | 743 | 6815 | 0.109 |
| **Subtotal** |  |  | **559** | **8181** | **0.064** | **372** | **8851** | **0.040** | **931** | **17591** | **0.053** |
| **Nonchronic disease** | < 29 | 20.0 | 158 | 23806 | 0.007 | 87 | 27616 | 0.003 | 245 | 51422 | 0.005 |
|  | 29 - 37 | 32.4 | 88 | 18742 | 0.005 | 40 | 22970 | 0.002 | 128 | 41712 | 0.003 |
|  | 37 - 46 | 40.7 | 142 | 16689 | 0.008 | 54 | 20416 | 0.003 | 196 | 37105 | 0.005 |
|  | 46 - 56 | 49.9 | 221 | 11767 | 0.018 | 93 | 14736 | 0.006 | 314 | 26503 | 0.012 |
|  | > 56 | 64.7 | 602 | 8002 | 0.070 | 321 | 7357 | 0.042 | 923 | 15359 | 0.060 |
| **Subtotal** |  |  | **1211** | **79006** | **0.015** | **595** | **93690** | **0.006** | **1806** | **173907** | **0.010** |
| **Respiratory**  (COPD, Asthma and Smoking) | < 29 | 15.2 | 15 | 3661 | 0.004 | 9 | 2440 | 0.004 | 24 | 6101 | 0.004 |
|  | 29 - 37 | 32.5 | 16 | 3468 | 0.005 | 5 | 2730 | 0.002 | 21 | 6198 | 0.003 |
|  | 37 - 46 | 40.9 | 16 | 2606 | 0.006 | 3 | 2301 | 0.001 | 19 | 4907 | 0.004 |
|  | 46 - 56 | 49.9 | 42 | 1692 | 0.024 | 12 | 1376 | 0.009 | 54 | 3068 | 0.018 |
|  | > 56 | 68.7 | 150 | 1415 | 0.096 | 60 | 791 | 0.071 | 210 | 2206 | 0.095 |
| **Subtotal** |  |  | **239** | **12842** | **0.018** | **89** | **9727** | **0.009** | **328** | **22808** | **0.014** |
| **Others** | < 29 | 22.9 | 37 | 617 | 0.057 | 25 | 778 | 0.031 | 62 | 1395 | 0.044 |
|  | 29 - 37 | 32.4 | 11 | 342 | 0.031 | 9 | 637 | 0.014 | 20 | 979 | 0.020 |
|  | 37 - 46 | 40.5 | 12 | 357 | 0.033 | 9 | 682 | 0.013 | 21 | 1039 | 0.020 |
|  | 46 - 56 | 49.8 | 22 | 297 | 0.069 | 10 | 497 | 0.020 | 32 | 794 | 0.040 |
|  | > 56 | 67.0 | 63 | 316 | 0.166 | 46 | 347 | 0.117 | 109 | 663 | 0.164 |
| **Subtotal** |  |  | **145** | **1929** | **0.070** | **99** | **3040** | **0.032** | **244** | **5114** | **0.048** |
| **Total** |  |  | **4391** | **132205** |  | **2767** | **149091** |  | **7158** | **283450** |  |
